# The *GADD45A* (1506T>C) Polymorphism Is Associated with Ovarian Cancer Susceptibility and Prognosis

**DOI:** 10.1371/journal.pone.0138692

**Published:** 2015-09-30

**Authors:** Cunzhong Yuan, Xiaoyan Liu, Xiaolin Liu, Ning Yang, Zhenping Liu, Shi Yan, Keng Shen, Beihua Kong

**Affiliations:** 1 Department of Obstetrics and Gynecology, Qilu Hospital of Shandong University, Ji'nan, Shandong, P.R. China; 2 Gynecologic Oncology Key Laboratory of Shandong Province, Qilu Hospital of Shandong University, Ji'nan, Shandong, P.R. China; 3 Department of Obstetrics and Gynecology, Peking Union Medical College Hospital, Chinese Academy of Medical Sciences and Peking Union Medical College, Beijing, P.R. China; China Medical University, TAIWAN

## Abstract

*GADD45A (*growth arrest and DNA damage 45 A) is the first stress-inducible gene identified to be a target of *p53*. However, no studies to date have assessed variants of the *GADD45* gene and their potential relationship to tumor susceptibility. We investigated the association of the *GADD45A* (1506T>C) polymorphism with ovarian cancer development in 258 ovarian cancer patients and 332 age-matched healthy women as controls using sequence analysis. We found a statistically significant difference in the *GADD45A* (1506T>C) genotype distributions between the case and control groups (TT vs. TC vs. CC, P = 0.0021) and found that variant 1506T>C was significantly associated with an increased risk of ovarian cancer (P<0.001, OR = 1.71, 95% CI [1.28–2.29]). We observed a statistically significant effect between tumor histology (P = 0.032) and CA125 status (P = 0.021). Carrying the C allele (TC+CC) was associated with an increased risk of positive CA125 (OR = 3.20, 95% CI [1.15–8.71). Carrying the T allele (TT+TC) showed a significant correlation with both higher *GADD45A* mRNA expression and longer ovarian cancer RFS (relapse-free survival) and OS (overall survival). We are the first group to demonstrate that the *GADD45A* (1506T>C) polymorphism is associated with ovarian cancer susceptibility and prognosis. These data suggest that *GADD45A* (1506T>C) is a new tumor susceptibility gene and could be a useful molecular marker for assessing ovarian cancer risk and for predicting ovarian cancer patient prognosis.

## Introduction

Ovarian cancer is the deadliest cancer of the female reproductive system, with over 21,980 new cases and over 14,270 deaths in the United States in 2014[1]. Similar to other malignancies, ovarian cancer occurs as a result of interactions between the environment and genetic factors.

An accumulation of genetic variants in many genes may be involved in the process of ovarian carcinogenesis [2]; for example, *BRCA1*, *BRCA2*, *RAD51C*, *RAD51D*, *MLH1*, *MSH2*, *RB1*, *CASP8*, *LIN28B*, *SMAD6*, *ERCC4* and PRG have been implicated in this process [3–12]. Recently, genome-wide association studies (GWAS) have found several common susceptibility alleles in four loci with strong associations [13–15]. Braem et al. reviewed 147 candidate genes and 3 GWAS published from 1990 to October 2010, including approximately 1100 genetic variants in more than 200 candidate genes and 20 intergenic regions [8]. However, only a few genetic variants exhibited strong evidence of an association with ovarian cancer, and the identification of genes associated with a predisposition to ovarian cancer requires further investigation [8].

The growth arrest and DNA damage 45 (*GADD45*) family consists of three members, *GADD45A*,-*B*, and-*G*. The human *GADD45A* gene is located on chromosome 1 (1p31.2–31.1), contains 4 exons and 3 introns, encodes a 165 amino acid acidic protein (18.4 kDa), and is highly conserved in all species [16]. As a confirmed target of p53 [17, 18], *GADD45A* can be regulated by both p53-dependent (ionizing radiation) and p53-independent (non-ionizing radiation) pathways [19] and plays important roles in the G2/M checkpoint and in genome stability [18]. Multiple transcription factors, such as *BRCA1*, *WT1*, *Oct-1*, *NF-YA*, *ATF4*, *AP-1*, *c-myc*, *ZBRK1*, and *Jun D*, can regulate *GADD45A* expression by binding to the intronic or promoter region at the transcriptional level [16, 18, 20, 21].

Previous studies have shown that downregulation of *GADD45A* and *GADD45G* enables tumor cells to escape programmed cell death in multiple tumor types [22]. *GADD45* expression is frequently decreased in non-small cell lung cancer [23], hepatocellular carcinoma [22], and glioblastoma [24]. The downregulation of *GADD45* expression confers poor tumor prognosis and is correlated with the differentiation status. Abnormal methylation of the *GADD45A* gene promoter region has been found in multiple breast cancer cell lines and breast cancer samples, but not in lymph nodes or normal mammary epithelium [25]. *GADD45A* overexpression is associated with a favorable prognosis, which suggests that the abnormal methylation of *GADD45* may contribute to breast cancer risk [25]. However, no *GADD45A* polymorphism associated with tumor susceptibility has been reported. A variant *GADD45A* (1506T>C) located at intronic regions was found in our study. Thus, we hypothesized that this variant might disrupt transcription factor binding sites, thereby altering *GADD45A* expression and affecting tumor genesis. Thus, we investigated the *GADD45A* (1506T>C) polymorphism, *GADD45A* expression, and ovarian cancer risk and prognosis.

## Materials and Methods

### Patients and Samples

This study included 258 patients diagnosed with ovarian cancer (mean age of 52.1± 13.9 years, from 21 to 81 years old) in Qilu Hospital (Shandong, China) between September 2008 and September 2012. Clinical characteristics, including age at diagnosis, degree of differentiation, FIGO stage, histological type, lymph node metastasis, CA125 status, and tumor size, were obtained from the patients’ medical records. In addition, 332 age-matched healthy women (mean age of 50.2 ± 13.4 years, from 21 to 82 years old) were recruited as controls from among patients undergoing a physical examination in our hospital. We calculated the *GADD45A* genotype with respect to relapse-free survival (RFS) and overall survival (OS) in 151 ovarian patients based on our ability to contact the patients.

Most of the subjects were of Han Chinese background and resided in Shandong Province, China. All of the participants provided written informed consent to participate in this study. The Ethical Committee of Shandong University approved this research (IRB number: KYLL-2013-019). All participants (patients and controls) donated 2 ml of peripheral blood, which was stored at -80°C in our laboratory.

DNA was extracted using a TIANamp Genomic DNA Kit (Tiangen, Beijing, China) according to the manufacturer’s protocol. The DNA concentration and purity were measured using an ultraviolet spectrophotometer (GE Healthcare, USA). The DNA samples were routinely stored at -80°C.

### Genotyping Analysis of GADD45A (1506T>C)

Genotyping of the *GADD45A* (1506T>C) polymorphism was performed using PCR and sequencing. The sequence of the *GADD45A* gene was obtained from NCBI (Gene ID: 1647, GenBank sequence, AY135686.1, GI: 22122007). Primers were designed using Primer Premier 5 according to the sequence of *GADD45A* as follows: forward primer 5'- AGTTTGCACAGGGCAACTCC-3' and reverse primer 5'- CCTGCTAAAGGAATTAGTCACG-3'. The PCR product size was 1255 bp. PCR amplification was performed in a final volume of 50 μL, containing 1 μL of genomic DNA (100 ng/μl), 4 μL of 2.5 mM dNTPs, 5 μL of buffer, 2 μL of each primer and 1 U of high fidelity Taq Polymerase (TransStart FastPfu Fly DNA Polymerase, Transgen, Beijing, China). The PCR amplification conditions were as follows: 94°C for 5 min, followed by 35 cycles of 94°C for 30 seconds, 61°C for 30 seconds, and 72°C for 2 min, and a final extension step of 72°C for 10 min. All sequencing was performed by BioSune Biotechnology Co., Ltd. (Shanghai, China), and the sequence data were analyzed using Chromas 2.31 and MegAlign 7.0 software.

### RNA Isolation, Reverse Transcription PCR, and Quantitative Real-Time PCR (QRT-PCR)

Total RNA was extracted from cancer tissues using TRIzol reagent (Invitrogen) based on the suggested protocol. Reverse transcription PCR was performed using a PrimeScript RT-PCR kit (TaKaRa, Dalian, China). QRT-PCR was performed using the Applied Biosystems 7900HT Real-time PCR System. The mRNA sequence of the *GADD45A* gene was obtained from NCBI (NM_001924.3 GI: 315075321). The following primers were used to amplify the *GADD45A* gene: 5'-GAAAGGGATGGATAAGGTGGG-3' and reverse primer 5'-CCTGGATCAGGGTGAAGTGGA-3'. GAPDH (forward primer 5'-GGGCTGCTTTTAACTCTGGTAAAG-3' and reverse primer 5'-CCATGGGTGGAATCATATTGG-3') was used as the internal control. The experiments were repeated three times to confirm the findings.

### Statistical Analysis

Statistical analyses were performed as previously described [26]. Hardy-Weinberg equilibrium was tested for all of the SNPs using a public web-based statistical tool (http://www.oege.org/software/hwe-mr-calc.shtml), and the threshold for disequilibrium was P <0.05. The allele and genotype distributions in the ovarian cancer group and the control group were compared using chi-square tests, and Fisher’s exact test was used when the one cell count was <5. The risk of ovarian cancer development was estimated as an odds ratio (OR) with a 95% confidence interval (CI) using unconditional logistic regression analysis. The patient survival rates were estimated using the Kaplan-Meier method. Multivariate analysis of prognostic factors was performed using Cox regression analysis. All p-values were calculated as two-sided, and the threshold for significance was P <0.05. The data were analyzed using SPSS (Statistical Package for the Social Sciences) 17.0 (SPSS, Chicago, Illinois, USA).

## Results

### Relationship between the GADD45A (1506T>C) Polymorphism and Ovarian Cancer Risk

**Fig 1:**
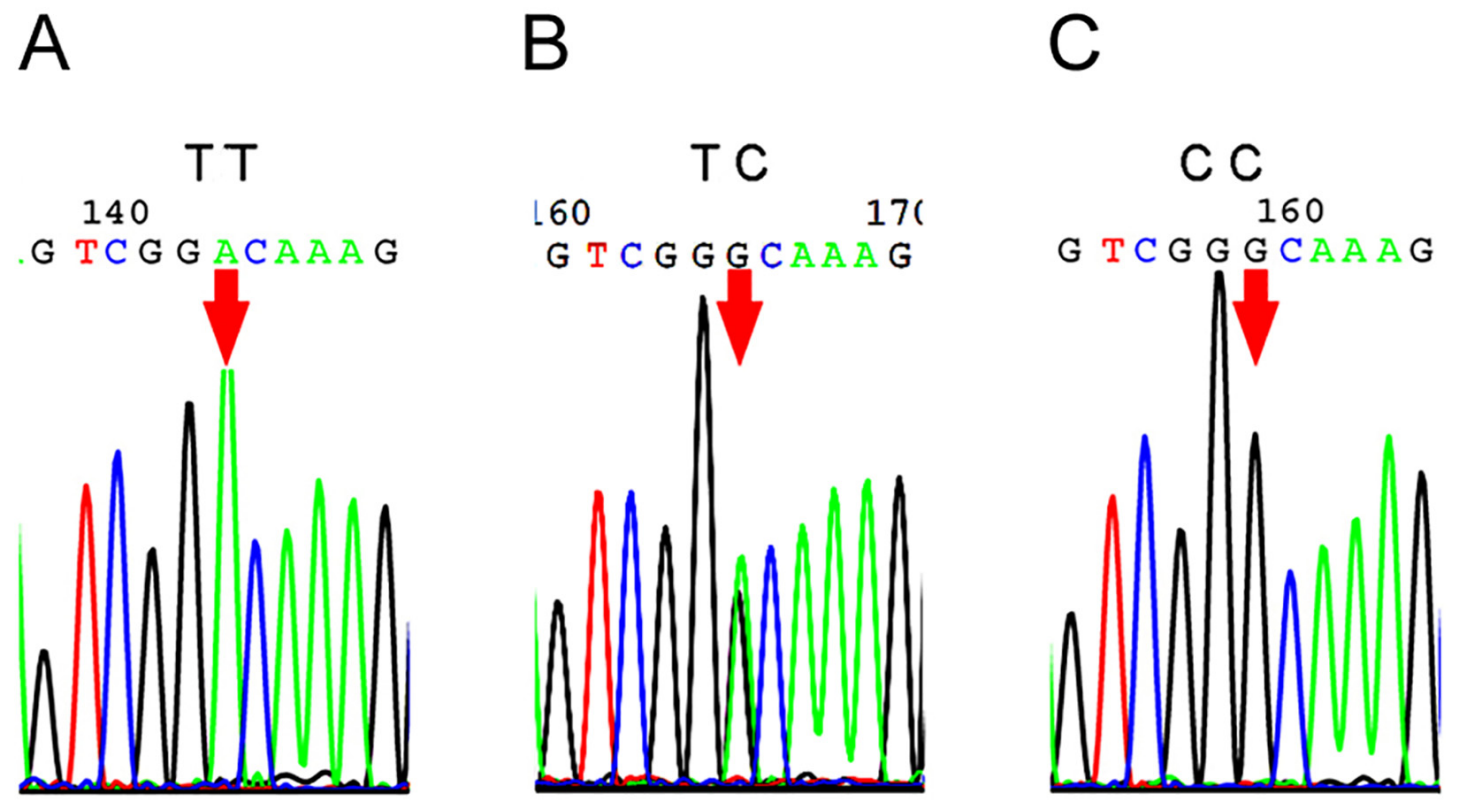
Sequencing chromatograms of *GADD45A (1506T>C)*. A-C, the reverse complement sequencing chromatogram results of the genotypes TT, TC and CC, respectively. The samples were randomly chosen.

SNP *GADD45A* (1506T>C) is a novel, previously unidentified variant. Sequencing chromatograms from randomly selected cases were used to illustrate variants of *GADD45A* (Fig 1).

The participants in the ovarian cancer and control groups were all from Mainland China; there were no significant clinical differences (i.e., median age, body mass index [BMI], menstrual history or other related parameters) between the 2 groups. Hardy-Weinberg equilibrium was tested. The chi-square values of the case group and control group were 0.51 and 0.47, respectively. As shown in Table 1, the *GADD45A* (1506T>C) genotypes and allele distributions exhibited a statistically significant difference between the case and control groups. Strong associations with ovarian cancer risk were found for SNP 1506T>C in the log-additive genetic model (TT vs. TC vs. CC, P = 0.0021), dominant genetic model (TT + TC vs. CC, P = 0.0017, OR = 1.95, 95% CI [1.28–2.95]), and recessive genetic model (TT vs. TC+CC, P = 0.0093, OR = 2.05, 95% CI [1.19–3.53]). We observed a greater prevalence of C alleles (P<0.001, OR = 1.71, 95% CI [1.28–2.29]) in ovarian cancer patients compared to the controls.

**Table 1:** The *GADD45A* (1506T>C) genotype and allele distribution in ovarian cancer and controls.

Genotype	Ovarian Cancer n(%)†	Controls n(%)†	P-value	OR 95%CI
TT	33(12.8)	77(23.1)	0.0021	1(reference)
TC	110(42.4)	158(47.5)		1.61(0.90–2.89)
CC	115(44.8)	97(29.4)		2.75(1.51–5.00)
TT+TC	143(55.2)	235(70.6)	0.0017	1(reference)
CC	115(44.8)	97(29.4)		1.95(1.28–2.95)
TT	33(12.8)	77(23.1)	0.0093	1(reference)
TC+CC	225(87.2)	255(76.9)		2.05(1.19–3.53)
T	176(35.0)	312(46.8)	<0.001	1(reference)
C	340(68.0)	352(53.2)		1.71(1.28–2.29)

† The X^2^ for Hardy-Weinberg equilibrium test results of the ovarian cancer group and the control group are 0.51 and 0.47, respectively (both *P* > 0.05).

In summary, variant 1506T>C is correlated with an increase in ovarian cancer risk.

### Relationship between the GADD45A (1506T>C) Polymorphism and Clinicopathological Variables

As shown in Table 2, there is an association of the TT and TC+CC genotypes with the clinicopathological characteristics, including age at diagnosis, degree of tumor differentiation, clinical stage, lymph node metastasis, CA125 expression, tumor size and tumor histology. We observed a statistically significance effect between tumor histology (P = 0.032) and CA125 status (P = 0.021). Carrying the C allele (TC+CC) was associated with an increased risk of positive CA125 (OR = 3.20, 95% CI [1.15–8.71).

**Table 2:** Results of association analysis between *GADD45A* (1506T>C) and the clinicopathological characteristics.

Clinical data information	All (%)	Genotype		P-value	OR 95%CI
		TT (%)	TC+CC (%)		
Age					
≤50	88(34.3)	15(5.8)	73(28.5)	0.238	1.00(reference)
>50	170(65.7)	18(7.0)	152(58.7)		0.582(0.235–1.440)
Degree of Differentiation					
Low	185(84.8)	23(10.3)	162(74.5)	0.677	1.00(reference)
Middle & High	33(15.2)	3(1.4)	30(13.8)		0.720(0.153–3.394)
Unknown	40				
Clinical stage					
I & II	69(28.9)	12(5.0)	57 (23.9)	0.177	1.00(reference)
III & IV	170(71.1)	17(6.9)	153 (64.2)		0.512(0.191–1.370)
Unknown	19				
Positive lymph node					
Negative	74(66.2)	12(10.8)	62(55.4)	0.540	1.00(reference)
Positive	38(33.8)	3(2.70)	35(31.1)		0.594(0.111–3.188)
Unknown	146				
CA125					
>65(U/ml)	212(84.0)	29(8.64)	183(75.3)	0.021	1.00(reference)
≤65(U/ml)	39(16.0)	11(4.32)	28(11.7)		3.20(1.15–8.71)
Unknown	7				
Size of tumor					
<10 cm	145(60.2)	16(6.8)	129(53.4)	0.608	1.00(reference)
≥10 cm	96(39.8)	14(5.6)	82(34.2)		1.279(0.498–3.287)
Unknown	17				
Tumor histology					
Serous	174(76.3)	15(6.6)	159(69.7)	0.032	1.00(reference)
Other	54(23.7)	16(7.0)	38 (16.7)		2.688(1.063–6.803)
Unknown	30				

### Relationship between the GADD45A (1506T>C) Polymorphism and GADD45A Expression Levels

**Fig 2:**
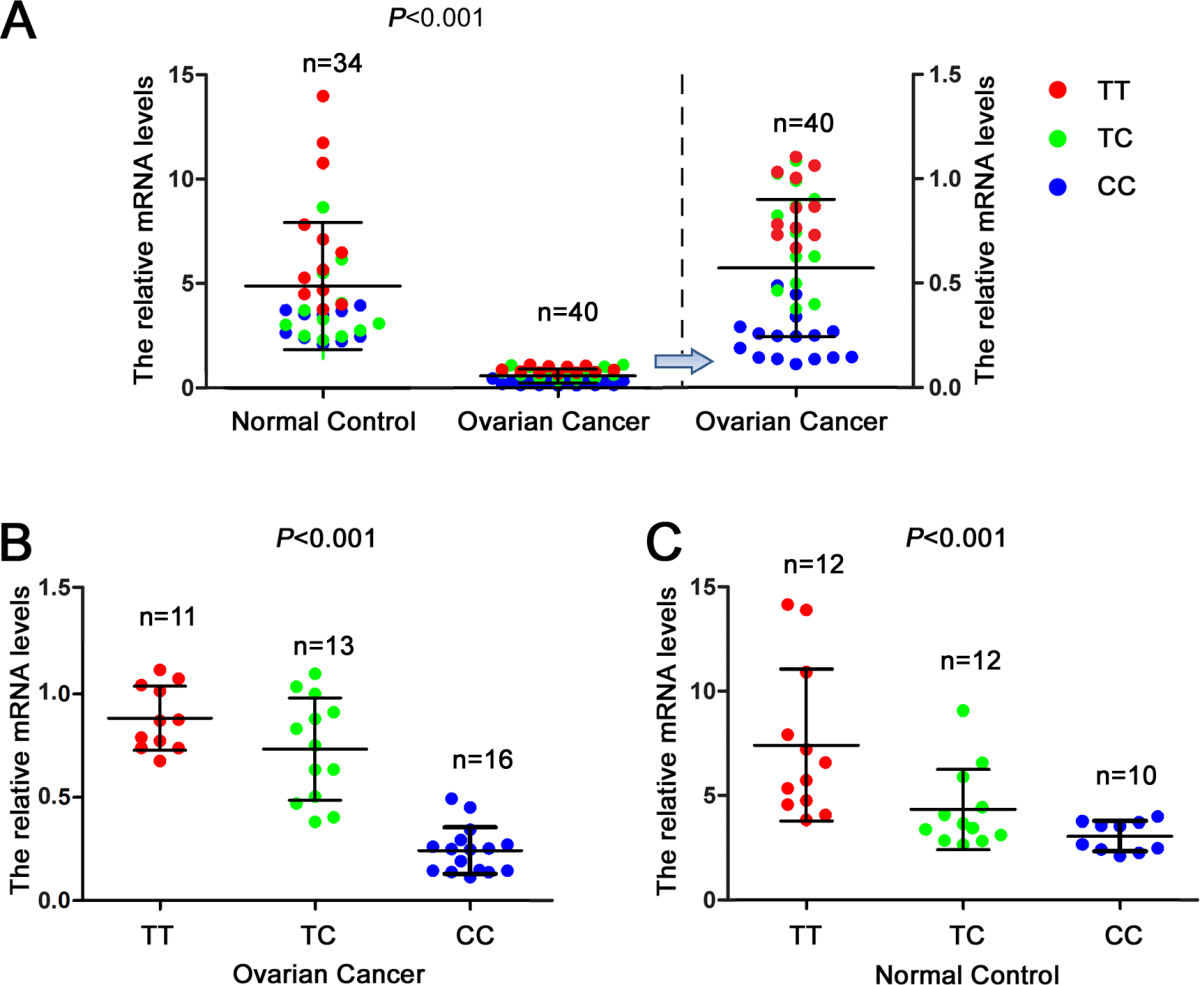
Association of the *GADD45A (1506T>C)* genotype and *GADD45A* mRNA expression. A, Relative levels of GADD45A mRNA expression in ovarian cancer tissues compared to normal ovarian tissues. B, Relative levels of GADD45A mRNA expression in the ovarian cancer tissues of patients with different 1506T>C genotypes. C, Relative levels of GADD45A mRNA expression in the normal control tissues of subjects with different 1506T>C genotypes. One spot represents the mean of three independent measurements obtained from one subject. Distributions of the three genotypes were random between the groups. N represents the number of samples in each respective group. Bars represent the standard deviation. Student’s *t*-test was used to evaluate the differences in the expression levels of different constructs.

We further tested the potential relationship between the *GADD45A* (1506T>C) polymorphism and *GADD45A* mRNA expression levels in vivo. As shown in Fig 2A, the levels of *GADD45A* mRNA in 22 ovarian cancer tissues were significantly lower than the levels observed in 15 normal tissues (P<0.001). As shown in Fig 2B, the *GADD45A* mRNA expression was higher in patients with TT or TC genotypes compared to patients with the CC genotype (P<0.001). As shown in Fig 2C, the *GADD45A* mRNA expression was higher in controls with TT or TC genotypes than with the CC genotype, (P<0.001). These findings suggest that the SNP 1506T>C may significantly affect the expression of the *GADD45A* gene.

### Relationship between GADD45A (1506T>C) Polymorphism and Ovarian Cancer Prognosis

**Fig 3:**
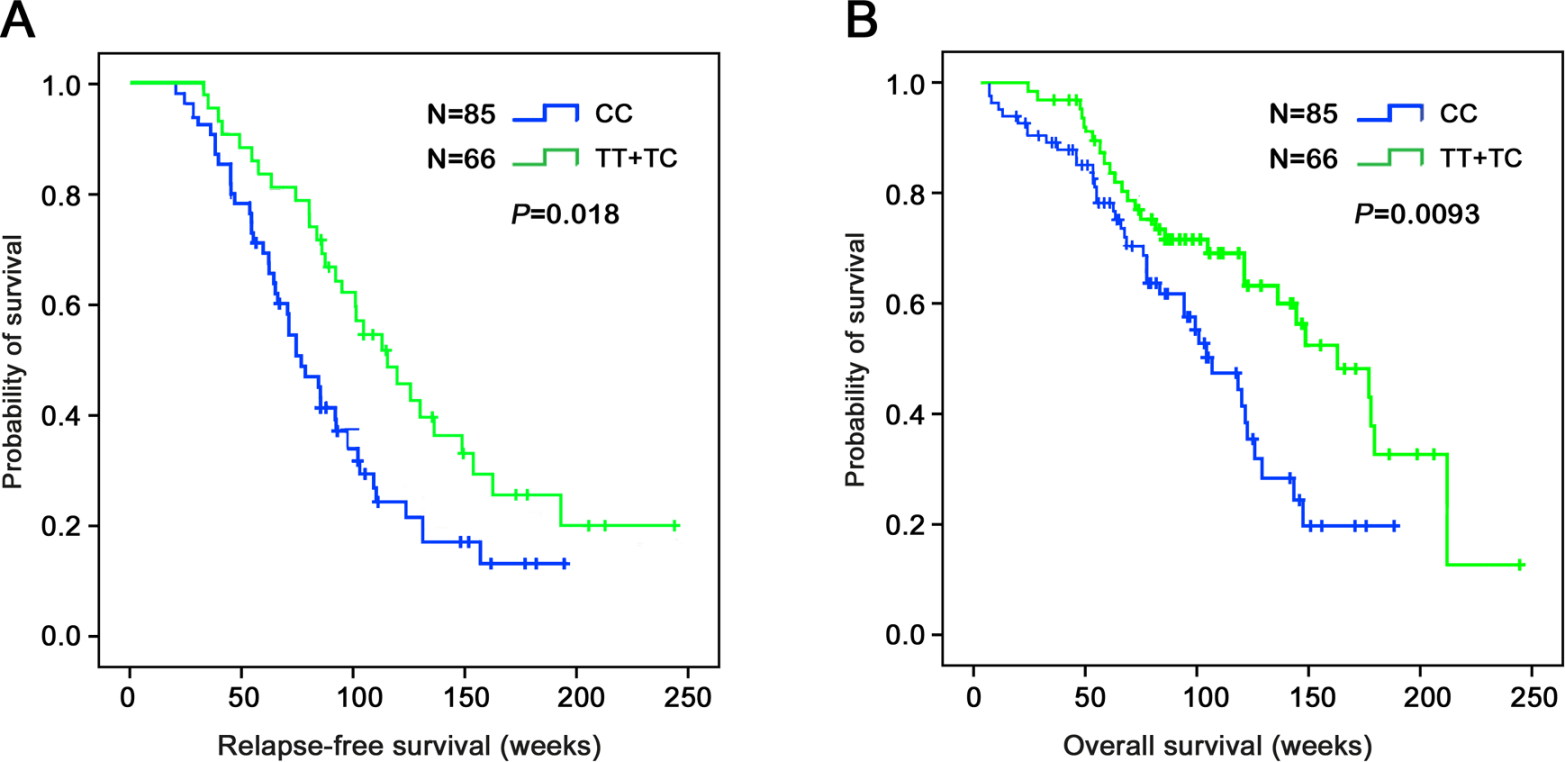
*GADD45A* genotypes and ovarian cancer prognosis. A, Genotype TT+TC of *GADD45A* had longer relapse-free survival (P = 0.018). B, Genotype TT+TC of *GADD45A* had longer overall survival (P = 0.0093).

To test the prognostic power of the *GADD45A* polymorphism in ovarian cancer, we calculated the *GADD45A* genotype with respect to ovarian relapse-free survival (RFS) and overall survival (OS). As shown in Fig 3, the TT+TC genotype showed significant correlation with both longer ovarian cancer RFS and OS in 151 patients (P = 0.018, P = 0.0093, respectively).

## Discussion

A large number of genetic variants in the candidate genes involved in the process of ovarian carcinogenesis have been reported [8]. Three GWAS with overlapping sets of study participants for ovarian cancer have been performed [13–15]. A total of 18 SNPs in 5 regions were found to be associated with ovarian cancer in 3 GWAS studies. However, there was no overlap between the genetic variants identified through the GWAS studies and the variants identified using a candidate gene search[8]. We believe that the evidence of an association with ovarian cancer is insufficient and that it is necessary to further investigate candidate genes associated with ovarian cancer.

*GADD45* expression is frequently decreased in some cancers [22–24]. Abnormal *GADD45* expression has been associated with tumor prognosis [25], and abnormal *GADD45* methylation may contribute to cancer risk [25]. A study showed that 1,25-dihydroxyvitamin D3 causes cell cycle arrest at the G2/M transition through p53-independent induction of GADD45 in ovarian cancer cells [27]. The induction of GADD45A expression might play a role in mediating the apoptotic response of ovarian cancer cells to the synthetic retinoid CD437 [28]. The association of *GADD45A* polymorphisms with tumor susceptibility and prognosis has never been reported.

In this study, the *GADD45A* (1506T>C) polymorphism was found to be associated with the ovarian cancer risk, clinicopathological characteristics, *GADD45A* expression levels and ovarian cancer prognosis. To the best of our knowledge, *GADD45A* is a novel ovarian cancer susceptibility gene and this study is the first to report an association between germline mutations in *GADD45A* and tumor risk and prognosis. We expect *GADD45A* to be a useful molecular marker for assessing ovarian cancer risk and prognosis.

The SNP 1506T>C is located in introns and thus cannot alter the sequence or structure of the protein. However, we understand that the variant 1506T>C was associated with and increased risk of ovarian cancer and the decreased survival of the patient. Furthermore, *GADD45A* functions downstream of *BRCA1* and *p53* in the DNA damage pathway. *GADD45A* expression is regulated by multiple transcription factors, such as *p53*, *BRCA1*, *WT1*, *Oct-1*, *NF-YA*, *ATF4*, *AP-1*, *c-myc*, and *ZBRK1*, which typically bind to the promoter or intronic regions of *GADD45A* [16, 20, 21, 29]. Thus, we hypothesized that this variant might disrupt transcription factor binding sites, thereby altering *GADD45A* expression and affecting tumor genesis. Consistent with findings obtained in other cancer studies [22–24], *GADD45A* mRNA expression was lower in ovarian patients than controls. Moreover, *GADD45A* mRNA expression was lower in patients with the CC genotype compared to patients carrying the T allele (TT+TC), and the survival of patients with the CC genotype was poorer. Thus, carrying the C allele (TC+CC) was associated with an increased risk of positive CA125. Those results were consistent with the function of *GADD45A*. *GADD45A* is implicated in active DNA demethylation, apart from the maintenance of genomic stability, DNA repair and suppression of cell growth [29,30].

As previously indicated, the participants involved in our study were mainly residents of Shandong Province, China. Because the Chinese population is generally more genetically homogeneous than other ethnic populations, we predict that these findings will be consistent in larger sample sizes across China, but determining the applicability of our findings to other ethnic populations (both within and outside Asia) requires further investigation in different patient populations, and larger sample sizes are required before these data can be extrapolated to other ethnicities [30].

In conclusion, we found, for the first time, that the *GADD45A* (1506T>C) polymorphism may be correlated with ovarian cancer susceptibility, clinicopathological characteristics, *GADD45A* expression levels and ovarian cancer prognosis. Because *GADD45A* has been demonstrated to be a key regulator in a complex network of oncogenic pathways, further investigations are needed to elucidate the functional role of this gene and its association with ovarian carcinogenesis and development. The variants of *GADD45A* may prove to be useful markers for the identification of ovarian cancer-susceptible patients or prognostic indicators of disease progression, and they may also serve as potential targets for future therapies.

In this study, for the first time, we demonstrate that the *GADD45A* (1506T>C) polymorphism is associated with ovarian cancer susceptibility and prognosis. A relationship was observed between the *GADD45A* (1506T>C) polymorphism and *GADD45A* expression levels. These data suggest that *GADD45A* (1506T>C) is a new tumor susceptibility gene and could be a useful molecular marker for assessing ovarian cancer risk and for predicting ovarian cancer patient prognosis.
